# Fatal Human Neurologic Infection Caused by Pigeon Avian Paramyxovirus-1, Australia

**DOI:** 10.3201/eid2912.230250

**Published:** 2023-12

**Authors:** Siobhan Hurley, John Sebastian Eden, John Bingham, Michael Rodriguez, Matthew J. Neave, Alexandra Johnson, Annaleise R. Howard-Jones, Jen Kok, Antoinette Anazodo, Brendan McMullan, David T. Williams, James Watson, Annalisa Solinas, Ki Wook Kim, William Rawlinson

**Affiliations:** Prince of Wales Hospital, Randwick, New South Wales, Australia (S. Hurley, K.W. Kim);; Westmead Institute for Medical Research Centre for Virus Research, Westmead, New South Wales, Australia (J.S. Eden);; Sydney Institute for Infectious Diseases, University of Sydney Faculty of Medicine and Health, Sydney, New South Wales, Australia (J.S. Eden, A.R. Howard-Jones);; CSIRO Australian Centre for Disease Preparedness, Geelong, Victoria, Australia (J. Bingham, M.J. Neave, D.T. Williams, J. Watson);; Prince of Wales and Sydney Children’s Hospital, Randwick (M. Rodriguez, A. Solinas);; Sydney Children’s Hospital, Randwick (A. Johnson, B. McMullan);; Centre for Infectious Diseases and Microbiology Laboratory Services, New South Wales Health Pathology–Institute of Clinical Pathology and Medical Research, Westmead (A.R. Howard-Jones, J. Kok);; Kids Cancer Centre, Sydney Children’s Hospital, Randwick (A. Anazodo);; University of New South Wales Faculty of Medicine and Health, School of Clinical Medicine, Sydney (B. McMullan, K. Kim);; Prince of Wales Hospital and Community Health Services, Sydney (W. Rawlinson);; University of New South Wales Schools of Clinical Medicine, Biotechnology and Biomolecular Sciences, Sydney (W. Rawlinson)

**Keywords:** avian paramyxovirus-1, metagenomics, Newcastle disease virus, zoonoses, brain, histopathology, pigeon paramyxovirus type 1, viruses, Australia

## Abstract

Avian paramyxovirus type 1 (APMV-1) is a virus of birds that results in a range of outcomes, from asymptomatic infections to outbreaks of systemic respiratory and neurologic disease, depending on the virus strain and the avian species affected. Humans are rarely affected; those who are predominantly experience mild conjunctivitis. We report a fatal case of neurologic disease in a 2-year-old immunocompromised child in Australia. Metagenomic sequencing and histopathology identified the causative agent as the pigeon variant of APMV-1. This diagnosis should be considered in neurologic conditions of undefined etiologies. Agnostic metagenomic sequencing methods are useful in such settings to direct diagnostic and therapeutic efforts.

Newcastle disease is a common and highly contagious zoonotic infection affecting wild and domestic birds globally; it is caused by virulent strains of avian paramyxovirus type 1 (APMV-1) (*1*,*2*). APMV-1 is a negative-sense, single-stranded RNA virus belonging to the *Orthoavulavirus* genus, *Paramyxoviridae* family (*1*). Strains of this virus are categorized into 2 classes, I and II, on the basis of their fusion protein sequence (*1*). Substantial variation in virulence exists among strains of AMPV-1; class I strains are classically avirulent, whereas class II strains can be avirulent or virulent. Class II genotype VI strains of APMV-1, known as pigeon avian paramyxovirus type 1 (PPMV-1), commonly have high pathogenicity; pigeons and doves are reservoirs (*3*,*4*).

Newcastle disease in birds can manifest with fatal central nervous system (CNS), respiratory, and digestive diseases, depending on tropism of the particular strain (*2*). Whereas AMPV-1 primarily affects birds, the virus can infect nonavian hosts, including primates (humans, monkeys), rabbits, and pigs (*1*). In humans, cases reported have typically manifested as mild conjunctivitis, although fatal cases have been reported (*1*). We report a fatal case of neurologic infection caused by PPMV-1 in a child in Australia.

## Case Report

A 2-year-old child with a history of infantile pre-B cell acute lymphoblastic leukemia (ALL) was admitted to hospital with nausea and vomiting after 3 weeks of upper respiratory tract symptoms. As part of the Associazione Italiana di Ematologia Oncologia Pediatrica and the Berlin-Frankfurt-Münster Acute Lymphoblastic Leukemia (AIEOP-BFM ALL) clinical trial, she had been randomized to receive blinatumomab, a bispecific T-cell–engaging antibody targeting CD19-expressing B cells; her last dose was 6 months before admission (*5*). The patient had completed her second cycle of reinduction chemotherapy with cytarabine, 6-mercaptopurine, and cyclophosphamide 6 weeks before she was brought for care. No recent travel, pets, or unwell contacts were reported. Her condition progressed over 4 days; she became febrile and experienced sudden-onset superrefractory status epilepticus in the setting of fever. The clinical syndrome was consistent with febrile infection-related epilepsy syndrome (FIRES) (*6*).

Results of initial cerebral magnetic resonance imaging (MRI) were unremarkable. Investigations for autoimmune causes of encephalitis all yielded negative results, including testing for antibodies to myelin oligodendrocyte glycoprotein aquaporin 4, N-methyl-d-aspartate (NMDA) receptor, contactin-associated protein-like 2 (CASPR2), leucine-rich glioma-inactivated 1 (LGI-1), gamma aminobutyric acid-B (GABA-B), dipeptidyl-peptidase-like protein 6 (DPPX), and immunoglobulin-like cell adhesion molecule 5 (IgLON5). Neurotropin was elevated in cerebrospinal fluid (CSF) at 1,752 nmol/L (reference range 6–30 nmol/L), representing significant CNS inflammation. Ultra-rapid exome sequencing performed by Acute Care Genomics, a tool that enables diagnosis of genetic disorders within 5 days, yielded no genetic abnormalities (*7*). Results of extensive culture and PCR-based investigations for infectious causes, including bacterial, viral, fungal, and mycobacterial pathogens, were all negative.

**Figure 1:**
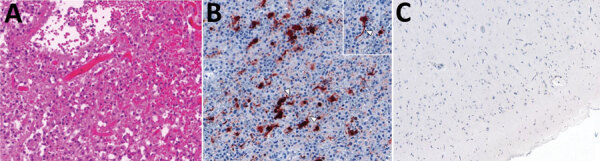
Histology of brain tissue and imaging from a fatal neurologic infection in an immunocompromised child in Australia that was caused by APMV-1. A) Brain biopsy showing extensive cortical necrosis with a dense infiltrate of macrophages. Hematoxylin and eosin stain; original magnification ×20. B) Immunohistochemistry of brain biopsy showing cytoplasmic APMV-1 nucleoprotein, probably in neurons, with axon-like processes (arrowheads). Original magnification ×20 (inset ×40). C) Immunohistochemistry of APMV-1 nucleoprotein, demonstrating the absence of immunolabeling in normal brain tissue. APMV, avian paramyxovirus.

Haematoxylin and eosin (H&E) staining of brain biopsy tissue performed 20 days after admission demonstrated almost complete cortical necrosis with some subpial sparing. The cortex was replaced by sheets of foamy macrophages with some gliosis and scattered, predominantly interstitial, CD3-positive T cells. Only rare residual small neurons were identified in the superficial cortex with NeuN immunostaining. No microglial nodules, viral inclusions, or viral cytopathic effects were apparent (Figure 1, panel A). No viral pathogens were present in a range of samples, including CSF, plasma, and brain tissue; relevant negative results were for HHV6, BK virus, SARS-CoV-2, parvovirus B19, herpes simplex viruses (HSV-1, HSV-2), varicella zoster virus, cytomegalovirus, Epstein-Barr virus, parainfluenza virus, adenovirus, human metapneumovirus, enterovirus, rhinovirus, bocavirus, coronavirus, and JC virus. Culture results for bacterial and fungal pathogens on those samples, panmycobacterial PCR, and 16S ribosomal RNA PCR performed on brain tissue were all negative.

**Figure 2:**
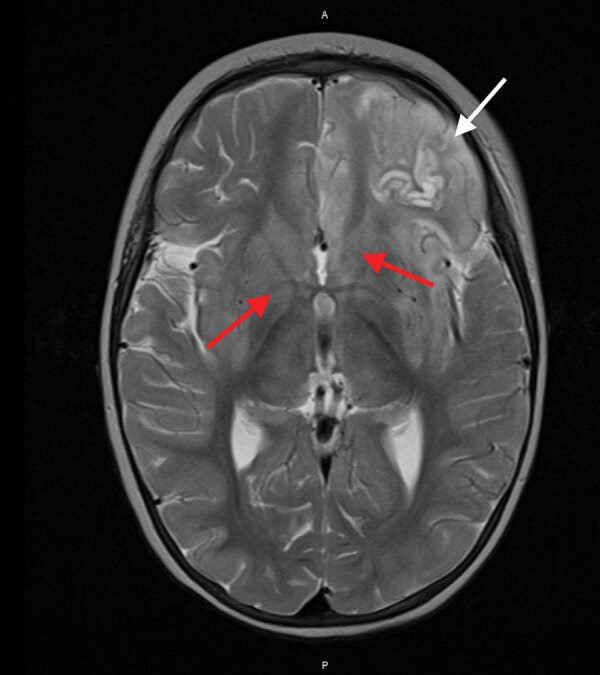
Magnetic resonance imaging of the brain of an immunocompromised child with avian paramyxovirus type 1 infection, Australia. Image, captured 16 days after hospital admission, shows predominantly left frontal and insular T2 signal hyperintensity evolving into laminar necrosis (white arrow) and hyperintensity of deep gray-matter structures (red arrows).

Despite therapy with broad-spectrum antimicrobial drugs, antiseizure medications, immunomodulators, and a ketogenic diet, the patient’s condition did not improve. Over the course of 2 weeks, serial cerebral MRI showed progressive and widespread inflammatory changes with increasing left frontal and insular T2 signal hyperintensity evolving into laminar necrosis as well as T2 hyperintensity of deep gray-matter structures (Figure 2). Treatment was withdrawn, and the child died 27 days after admission. A postmortem examination was not performed.

We conducted comprehensive, hypothesis-free metagenomic testing of the biopsied brain tissue using established panviral hybridization-capture (*8*) and unbiased metatranscriptomic (*9*) sequencing methods performed in parallel. After sequence quality filtering, host nucleic acid removal, and alignment to the National Center for Biotechnology Information nonredundant/nucleotide database, both approaches independently identified APMV-1 as the dominant nonhuman sequences. Hybrid capture methods identified 39,928 APMV-1 sequences using Twist Biosciences viral panel (https://www.twistbioscience.com/resources/product-sheet/twist-pan-viral-panel) and 281,601 sequences by in-house methods. The only other nonhuman sequences of note included low levels (<230 reads) of human pegivirus (HPgV) sequences identified using both methods, as previously identified in similar studies (data not shown). 

**Figure 3:**
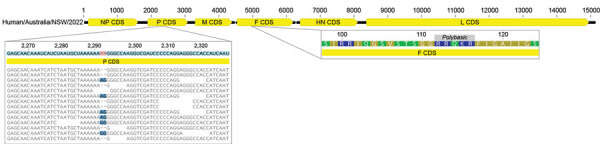
Genomic markers of virulence in avian paramyxovirus type 1 strain in an immunocompromised child in Australia. An analysis of both P and F genes indicated the strain would likely be classified as virulent based on P-gene editing; possible alternate reading frames were detected in mapped sequence reads (left side of panel). The F gene protein sequence carries a polybasic cleavage site at amino acid positions 112–116 (region on right of panel). CDS, coding sequences; F, fusion protein; G, glycoprotein; HN, hemagglutinin-neuraminidase protein; L, large protein; M, matrix protein; NP, nucleoprotein; P, polymerase-associated phosphoprotein.

**Figure 4:**
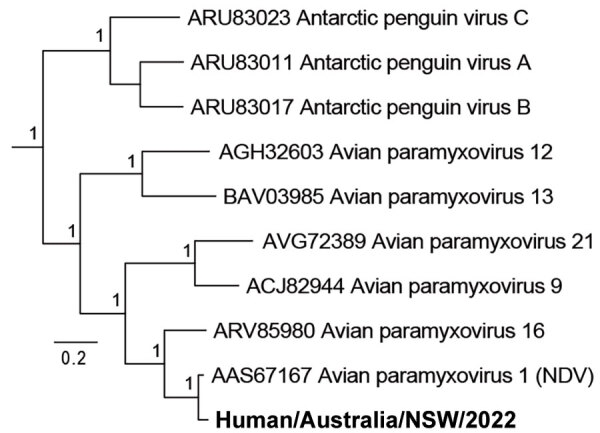
Phylogenetic classification of avian paramyxovirus type 1 strain from an immunocompromised child in Australia (bold) using the large polymerase protein sequence. Node support values show Shimodaira-Hasegawa—like approximate likelihood ratio test statistics with branch lengths proportional to the scale. GenBank accession numbers are provided for reference sequences. Scale bar indicates number of substitutions per site.

**Figure 5:**
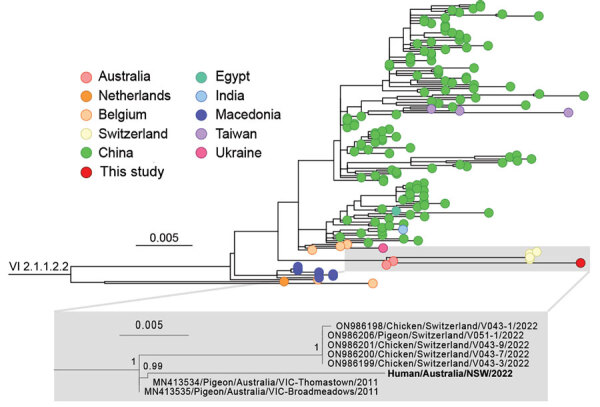
Phylogeny of avian paramyxovirus type 1 strain from an immunocompromised child in Australia (bold). Tree was prepared from MAFFT-aligned fusion gene sequences of genotype VI sublineage 2.1.1.2.2 strains (classified as pigeon avian paramyxovirus 1) using PhyML with the Hasegawa-Kishino-Yano + gamma DNA substitution model and rooted with a genotype VI sublineage 2.1.1.2.1 outgroup (HM063425/Pigeon/CHN/P4). Red dot indicates virus from this case; GenBank accession numbers are provided for reference sequences. Gray box indicates the branch containing the virus we identified, expanded to show detail. Colored circles at tips indicate country of sampling. The virus from our study appears to be related to viruses circulating in Australia since at least 2011. Node support values show Shimodaira-Hasegawa—like approximate likelihood ratio test statistics; branch lengths are proportional to the scale of the number of substitutions per site. Scale bars indicate number of substitutions per site. All strains used in this analysis are listed in the Appendix.

We performed de novo assembly of the nonhuman sequences from the brain metatranscriptomic library, which yielded a complete APMV-1 genome with a mean read depth of 2,568×. Comparative analysis of potential virulence markers identified evidence of P-gene editing and a polybasic F gene cleavage site, suggestive of a virulent (velogenic) strain (Figure 3). The avian paramyxovirus detected in this case was classified as a type-1 APMV species using a phylogenetic approach based on the L-protein sequence, in accordance with International Committee on Taxonomy of Viruses guidelines (*10*). Specifically, we aligned the complete large (L) protein sequences of reference and case viruses using MAFFT version 7 (https://mafft.cbrc.jp/alignment/software) before phylogenetic analysis with PhyML version 3.3.20180214 (www.atgc-montpellier.fr/phyml) using the Le-Gascuel (LG) protein substitution model (Figure 4). An analysis of the F gene (Figure 4) indicated the virus belonged to a putative Australian lineage of the pigeon variant, PPMV-1, belonging to class II, genotype VI, sublineage 2.1.1.2.2 (Figure 5). The complete genome sequence was submitted to GenBank (accession no. OR636618). 

APMV-1 infection of the brain tissue was confirmed using virus-specific real-time quantitative PCR assays (data not shown) and immunohistochemistry at the Australian Centre for Disease Preparedness. The APMV-1 nucleoprotein identified using immunohistochemistry was present in occasional clusters within cells in the brain tissue. At least some of those small pyramidal cells appeared to be neurons with an axon-like process (Figure 1, panel B). No staining was identified in appropriate negative controls, which were healthy juvenile brain and lymphoid (tonsil) tissue (Figure 1, panel C).

## Discussion

The first documented case of APMV-1 infection in humans was reported in 1942 in Australia (*1*). Since then, an additional 485 human cases have been reported globally, most (288 cases) in the United Kingdom (*1*). Most infections have been mild and self-limiting, commonly presenting as conjunctivitis (*1*). There have been 4 recorded human deaths, all caused by the PPMV-1 strain, in the Netherlands, United States, China, and France (*1*–*3*,*11*); 1 case-patient who died had a clear exposure to pigeons (*11*). Case-patients in the Netherlands, United States, and China all experienced respiratory symptoms and died from respiratory failure (*3*,*11*). Two of those case-patients were immunosuppressed with an underlying hematologic malignancy (*1*,*3*). The fourth, with marked similarities to the case we describe, involved a young girl who died following the sudden onset of progressive seizures, 3 months after hematopoietic stem cell transplantation for combined immunodeficiency (*2*). Those cases highlight the virulence of PPMV-1 and the potential for severe disease in contrast to other APMV-1 genotypes.

Although we detected a small number of HPgV reads (<230 reads), there is no clear association between HPgV and human disease, nor evidence of neurotropism. Because HPgV has a reported seroprevalence of up to 20% in the human population, this finding most likely represents passive transport into CSF (*9*). In the absence of other co-infecting viral, bacterial, or fungal pathogens, or other major organ pathology such as pneumonia, the child’s death was most likely caused by encephalitis from overwhelming PPMV-1 CNS infection.

In birds, APMV-1 infects respiratory, neural, lymphoid, and other tissues, causing a range of signs that are partially dependent on the viral strain. In the human case we report, it is likely that the PPMV-1 infection began in the upper respiratory tract before the onset of FIRES and spread to the CNS, given the history of respiratory signs. Although no exposure was identified, it is likely the virus was transmitted inadvertently via direct contact with pigeon feces or infected fluids. The virus is known to remain stable in pigeon feces and can be spread by windborne dust, extending the risk beyond just localized environments (*3*). In the absence of seroprevalence data of PPMV-1 in humans, the relationship between exposure and development of disease remains unknown and worthy of further investigation.

The term FIRES refers to an epileptic encephalopathy characterized by intractable seizures after a febrile illness. It is diagnosed in the absence of infectious encephalitis or a defined trigger and most commonly affects otherwise healthy children and young adults (*6*). FIRES has a high rate of sequelae; up to two thirds of surviving patients experience some degree of cognitive impairment. Severity ranges from mild intellectual disability to vegetative states; almost all patients have refractory seizures (*6*). The mortality rate is not insignificant, 10% in the acute phase and 13% in chronic phase (*6*). In cases in which a cause is identified, autoimmune pathologies predominate, followed by cerebral viral infection. An association between avian viruses and FIRES has not been documented as of October 2023.

The use of APMV-1 strains as an oncolytic therapy has been examined in preclinical and clinical trials; the favorable properties of APMV-1 result in an antitumor response (*12*). The strains used for head and neck squamous cell carcinoma (ATV-NDV), colorectal cancer (ATV-NDV-aHN-aCD2), and glioblastoma (NDV-HUJ) have shown promising results with no adverse effects (*12*). Although those strains in use belong to class I and are of low virulence, future exploration of APMV-1 as an oncolytic agent should consider the underlying virulence of genotypes and, although rare, the potential for devastating off-target effects such as CNS infections if PPMV-1 strains are used.

This case demonstrates the valuable role of metagenomics in complex clinical cases and highlights its potential future use in routine diagnostics. Although early diagnosis of PPMV-1 in this case was unlikely to have changed the clinical trajectory, including metagenomics early in similar cases is likely to have a substantial effect on patient outcomes. Compared with traditional methods, metagenomics can detect uncommon and novel pathogens in different disease phenotypes, which is particularly relevant in immunocompromised patients for whom a broad range of opportunistic pathogens must be considered (*13*,*14*). Metagenomics is of particular benefit compared with targeted (closed diagnosis) nucleic acid testing, such as PCR, that relies on a priori knowledge of an organism in the interrogation of tissue (*13*). Metagenomic methods enable all known pathogens to be tested in a single sample, which avoids the time delays associated with a traditional stepwise approach to diagnosis (*13*). The ability to test samples for such a breadth of organisms also conserves sample volume for difficult-to-obtain sample types such as cerebral tissue. From a laboratory workflow perspective, this approach could simplify diagnostics, avoiding the need to develop, validate and verify individual assays for each novel pathogen.

Incorporating metagenomic sequencing into routine diagnostics has several barriers and challenges. Metagenomic sequencing is expensive. High-throughput sequencing requires highly skilled staff, including bioinformaticians with expertise in data analysis (*15*); however, the use of robust cloud-based metagenomic sequence classification software tools could overcome that limitation (*16*). The potential for low-cost, robust testing with reduced local bioinformatic expertise requirements may enable lower-income and middle-income countries to use metagenomics as a diagnostic method, which would provide tertiary-level diagnostic abilities in a greater range of clinical settings. Care in handling samples is of utmost importance to avoid contamination and nucleic acid degradation that can affect the interpretation of results (*15*). As with conventional PCR, results should be interpreted in the appropriate clinical context (*15*). Incorporating pathogen-agnostic metagenomic testing into routine laboratory workflows improves laboratory diagnostics and our response to novel emerging disease threats, including emergent zoonoses. Improved diagnosis and monitoring of conditions with unknown etiology, such as FIRES, using metagenomic analyses has the potential to uncover novel and emergent pathogens, which expands the breadth of infectious-disease diagnosis. Such advances will also increase the potential for future targeted treatments and improved outcomes for children and adults with neurologic infections.

Appendix Additional information for case of fatal human neurologic infection caused by pigeon avian paramyxovirus-1, Australia.
