# Supplementary material for: Fatal Human Neurologic Infection Caused by Pigeon Avian Paramyxovirus-1, Australia
Source: Emerg Infect Dis. 2023 Dec;29(12):2482–7. doi: 10.3201/eid2912.230250 (PMC10683822; doi:10.3201/eid2912.230250)
Supplement: Appendix — Additional information for case of fatal human neurologic infection caused by pigeon avian paramyxovirus-1, Australia. [file 23-0250-Techapp-s1.pdf]

Article DOI: http://doi.org/10.3201/eid2912.230250

EID cannot ensure accessibility for supplementary materials supplied by authors. Readers who have difficulty accessing supplementary content should contact the authors for assistance.

Fatal Human Neurologic Infection Caused by Pigeon Avian Paramyxovirus-1, Australia

Appendix

Appendix Table. Source and metadata of sequences used for fusion gene analysis

Accession no.	Host	Country	Strain	Year	Lineage	Label
GenBank	Human	AUS	Sydney	2022	VI 2.1.1.2.2	GenBank/Human/AUS/Sydney/2022
JN872162	Rosella	BEL	4940	2008	VI 2.1.1.2.2	JN872162/Rosella/BEL/4940/2008
JQ979176	Pigeon	CHN	SDLC	2011	VI 2.1.1.2.2	JQ979176/Pigeon/CHN/SDLC/2011
JQ993431	Pigeon	CHN	SDS	2011	VI 2.1.1.2.2	JQ993431/Pigeon/CHN/SDS/2011
JX486552	Pigeon	CHN	110713	2011	VI 2.1.1.2.2	JX486552/Pigeon/CHN/110713/2011
JX901122	Pigeon	BEL	11-07574	2011	VI 2.1.1.2.2	JX901122/Pigeon/BEL/11-07574/2011
JX901123	Pigeon	BEL	11-08304	2011	VI 2.1.1.2.2	JX901123/Pigeon/BEL/11-08304/2011
JX901124	Pigeon	BEL	11-09620	2011	VI 2.1.1.2.2	JX901124/Pigeon/BEL/11-09620/2011
KJ600778	Pigeon	CHN	SH	2013	VI 2.1.1.2.2	KJ600778/Pigeon/CHN/SH/2013
KJ600779	Pigeon	CHN	SH	2013	VI 2.1.1.2.2	KJ600779/Pigeon/CHN/SH/2013
KJ607165	Pigeon	CHN	131237	2013	VI 2.1.1.2.2	KJ607165/Pigeon/CHN/131237/2013
KJ607166	Pigeon	CHN	131011	2013	VI 2.1.1.2.2	KJ607166/Pigeon/CHN/131011/2013
KJ782376	Pigeon	CHN	0707	2011	VI 2.1.1.2.2	KJ782376/Pigeon/CHN/0707/2011
KJ808819	Pigeon	CHN	BJ2013	2013	VI 2.1.1.2.2	KJ808819/Pigeon/CHN/BJ2013/2013
KM374056	Pigeon	CHN	1111	2013	VI 2.1.1.2.2	KM374056/Pigeon/CHN/1111/2013
KM374057	Pigeon	CHN	2365	2012	VI 2.1.1.2.2	KM374057/Pigeon/CHN/2365/2012
KM374058	Pigeon	CHN	2369	2012	VI 2.1.1.2.2	KM374058/Pigeon/CHN/2369/2012
KM374060	Pigeon	CHN	215	2011	VI 2.1.1.2.2	KM374060/Pigeon/CHN/215/2011
KM374061	Pigeon	CHN	2036	2012	VI 2.1.1.2.2	KM374061/Pigeon/CHN/2036/2012
KP861633	Pigeon	CHN	SD069	2012	VI 2.1.1.2.2	KP861633/Pigeon/CHN/SD069/2012
KT163261	Pigeon	CHN	0163	2012	VI 2.1.1.2.2	KT163261/Pigeon/CHN/0163/2012
KT163262	Pigeon	CHN	0167	2013	VI 2.1.1.2.2	KT163262/Pigeon/CHN/0167/2013
KT163263	Pigeon	CHN	0168	2013	VI 2.1.1.2.2	KT163263/Pigeon/CHN/0168/2013
KU522142	Pigeon	EGY	VRLCU	2014	VI 2.1.1.2.2	KU522142/Pigeon/EGY/VRLCU/2014
KU527559	Pigeon	CHN	DH09	2015	VI 2.1.1.2.2	KU527559/Pigeon/CHN/DH09/2015
KU527560	Pigeon	CHN	NG05	2015	VI 2.1.1.2.2	KU527560/Pigeon/CHN/NG05/2015
KX710210	Pigeon	IND	D168	2015	VI 2.1.1.2.2	KX710210/Pigeon/IND/D168/2015
KY788663	Grey_heron	CHN	GZ333	2015	VI 2.1.1.2.2	KY788663/Grey_heron/CHN/GZ333/2015
KY788667	European_turtle_dove	CHN	GZ23	2015	VI 2.1.1.2.2	KY788667/European_turtle_dove/CHN/GZ23/2015

Accession no.	Host	Country	Strain	Year	Lineage	Label
MF580815	Pigeon	CHN	GX0094	2011	VI 2.1.1.2.2	MF580815/Pigeon/CHN/GX0094/2011
MF580816	Pigeon	CHN	GX0001	2012	VI 2.1.1.2.2	MF580816/Pigeon/CHN/GX0001/2012
MF580817	Pigeon	CHN	GX0012	2012	VI 2.1.1.2.2	MF580817/Pigeon/CHN/GX0012/2012
MF580818	Pigeon	CHN	GX0019	2012	VI 2.1.1.2.2	MF580818/Pigeon/CHN/GX0019/2012
MF580819	Pigeon	CHN	GX0022	2012	VI 2.1.1.2.2	MF580819/Pigeon/CHN/GX0022/2012
MF580820	Pigeon	CHN	GX0029	2012	VI 2.1.1.2.2	MF580820/Pigeon/CHN/GX0029/2012
MF580821	Pigeon	CHN	GX0031	2012	VI 2.1.1.2.2	MF580821/Pigeon/CHN/GX0031/2012
MF580823	Pigeon	CHN	GX0505	2012	VI 2.1.1.2.2	MF580823/Pigeon/CHN/GX0505/2012
MF580824	Pigeon	CHN	GX0004	2013	VI 2.1.1.2.2	MF580824/Pigeon/CHN/GX0004/2013
MF580825	Pigeon	CHN	GX0119	2015	VI 2.1.1.2.2	MF580825/Pigeon/CHN/GX0119/2015
MF580826	Pigeon	CHN	GX0126	2015	VI 2.1.1.2.2	MF580826/Pigeon/CHN/GX0126/2015
MF580827	Pigeon	CHN	GX1103	2015	VI 2.1.1.2.2	MF580827/Pigeon/CHN/GX1103/2015
MG840651	Environmental	CHN	HD240	2017	VI 2.1.1.2.2	MG840651/Environmental/CHN/HD240/2017
MG840652	Pigeon	CHN	1233	2014	VI 2.1.1.2.2	MG840652/Pigeon/CHN/1233/2014
MG840653	Pigeon	CHN	1050	2017	VI 2.1.1.2.2	MG840653/Pigeon/CHN/1050/2017
MG840654	Pigeon	CHN	2068	2016	VI 2.1.1.2.2	MG840654/Pigeon/CHN/2068/2016
MG840655	Pigeon	CHN	1325	2017	VI 2.1.1.2.2	MG840655/Pigeon/CHN/1325/2017
MG840656	Pigeon	CHN	1344	2017	VI 2.1.1.2.2	MG840656/Pigeon/CHN/1344/2017
MG840657	Pigeon	CHN	2045	2014	VI 2.1.1.2.2	MG840657/Pigeon/CHN/2045/2014
MG840658	Pigeon	CHN	1205	2015	VI 2.1.1.2.2	MG840658/Pigeon/CHN/1205/2015
MG840659	Pigeon	CHN	1336	2015	VI 2.1.1.2.2	MG840659/Pigeon/CHN/1336/2015
MG840660	Pigeon	CHN	1453	2017	VI 2.1.1.2.2	MG840660/Pigeon/CHN/1453/2017
MH807446	Pigeon	CHN	BJ-01	2001	VI 2.1.1.2.2	MH807446/Pigeon/CHN/BJ-01/2001
MK335466	Pigeon	CHN	LNPPMV17	2017	VI 2.1.1.2.2	MK335466/Pigeon/CHN/LNPPMV17/2017
MK469964	Pigeon	CHN	GXG20	2015	VI 2.1.1.2.2	MK469964/Pigeon/CHN/GXG20/2015
MK469965	Pigeon	CHN	GXG29	2016	VI 2.1.1.2.2	MK469965/Pigeon/CHN/GXG29/2016
MK469967	Pigeon	CHN	GXG33	2016	VI 2.1.1.2.2	MK469967/Pigeon/CHN/GXG33/2016
MK469968	Pigeon	CHN	GXG35	2017	VI 2.1.1.2.2	MK469968/Pigeon/CHN/GXG35/2017
MK469970	Pigeon	CHN	GXG3	2012	VI 2.1.1.2.2	MK469970/Pigeon/CHN/GXG3/2012
MK469971	Pigeon	CHN	GXG6	2013	VI 2.1.1.2.2	MK469971/Pigeon/CHN/GXG6/2013
MK469973	Pigeon	CHN	GXG13	2014	VI 2.1.1.2.2	MK469973/Pigeon/CHN/GXG13/2014
MK516200	Pigeon	CHN	SD01	2013	VI 2.1.1.2.2	MK516200/Pigeon/CHN/SD01/2013
MK516201	Pigeon	CHN	SD03	2013	VI 2.1.1.2.2	MK516201/Pigeon/CHN/SD03/2013
MK749297	Pigeon	CHN	GXG1	2012	VI 2.1.1.2.2	MK749297/Pigeon/CHN/GXG1/2012
MK749298	Pigeon	CHN	GXG22	2015	VI 2.1.1.2.2	MK749298/Pigeon/CHN/GXG22/2015
MK749299	Pigeon	CHN	GXG24	2015	VI 2.1.1.2.2	MK749299/Pigeon/CHN/GXG24/2015
MK749300	Pigeon	CHN	GXG25	2015	VI 2.1.1.2.2	MK749300/Pigeon/CHN/GXG25/2015
MK749301	Pigeon	CHN	GXG28	2016	VI 2.1.1.2.2	MK749301/Pigeon/CHN/GXG28/2016
MK749302	Pigeon	CHN	GXG31	2016	VI 2.1.1.2.2	MK749302/Pigeon/CHN/GXG31/2016
MK764391	Chicken	CHN	HB-1	2015	VI 2.1.1.2.2	MK764391/Chicken/CHN/HB-1/2015
MK764392	Pigeon	CHN	TJ-1	2015	VI 2.1.1.2.2	MK764392/Pigeon/CHN/TJ-1/2015
MK896851	Pigeon	CHN	SD0315	2018	VI 2.1.1.2.2	MK896851/Pigeon/CHN/SD0315/2018
MN413534	Pigeon	AUS	Thomastown	2011	VI 2.1.1.2.2	MN413534/Pigeon/AUS/Thomastown/2011
MN413535	Pigeon	AUS	Broadmeadows	2011	VI 2.1.1.2.2	MN413535/Pigeon/AUS/Broadmeadows/2011
MN632520	Pigeon	TWN	AHRI107	2016	VI 2.1.1.2.2	MN632520/Pigeon/TWN/AHRI107/2016
MN632521	Pigeon	TWN	AHRI111	2017	VI 2.1.1.2.2	MN632521/Pigeon/TWN/AHRI111/2017
MN700940	Pigeon	CHN	YQ	2012	VI 2.1.1.2.2	MN700940/Pigeon/CHN/YQ/2012
MN862505	Pigeon	CHN	3P3	2018	VI 2.1.1.2.2	MN862505/Pigeon/CHN/3P3/2018
MN862506	Pigeon	CHN	190610-2	2019	VI 2.1.1.2.2	MN862506/Pigeon/CHN/190610-2/2019

Accession no.	Host	Country	Strain	Year	Lineage	Label
MN893303	Pigeon	CHN	GZ08	2017	VI 2.1.1.2.2	MN893303/Pigeon/CHN/GZ08/2017
MN893304	Pigeon	CHN	SZ12	2018	VI 2.1.1.2.2	MN893304/Pigeon/CHN/SZ12/2018
MN893305	Pigeon	CHN	HY25	2019	VI 2.1.1.2.2	MN893305/Pigeon/CHN/HY25/2019
MN953614	Pigeon	CHN	2453	2018	VI 2.1.1.2.2	MN953614/Pigeon/CHN/2453/2018
MN953615	Pigeon	CHN	2364	2018	VI 2.1.1.2.2	MN953615/Pigeon/CHN/2364/2018
MN953616	Pigeon	CHN	1427	2019	VI 2.1.1.2.2	MN953616/Pigeon/CHN/1427/2019
MN953617	Pigeon	CHN	1024	2019	VI 2.1.1.2.2	MN953617/Pigeon/CHN/1024/2019
MN953618	Pigeon	CHN	1433	2019	VI 2.1.1.2.2	MN953618/Pigeon/CHN/1433/2019
MT161598	Pigeon	CHN	GX0019	2012	VI 2.1.1.2.2	MT161598/Pigeon/CHN/GX0019/2012
MT811572	Pigeon	CHN	ZJ-01	2013	VI 2.1.1.2.2	MT811572/Pigeon/CHN/ZJ-01/2013
MT811573	Pigeon	CHN	ZJ-02	2013	VI 2.1.1.2.2	MT811573/Pigeon/CHN/ZJ-02/2013
MT811575	Pigeon	CHN	JS-01	2015	VI 2.1.1.2.2	MT811575/Pigeon/CHN/JS-01/2015
MT811576	Pigeon	CHN	JS-03	2015	VI 2.1.1.2.2	MT811576/Pigeon/CHN/JS-03/2015
MT811577	Pigeon	CHN	AH-06	2016	VI 2.1.1.2.2	MT811577/Pigeon/CHN/AH-06/2016
MT811578	Pigeon	CHN	JS-09	2016	VI 2.1.1.2.2	MT811578/Pigeon/CHN/JS-09/2016
MT811581	Pigeon	CHN	GS-12	2017	VI 2.1.1.2.2	MT811581/Pigeon/CHN/GS-12/2017
MT811582	Pigeon	CHN	JS-05	2019	VI 2.1.1.2.2	MT811582/Pigeon/CHN/JS-05/2019
MW147625	Pigeon	CHN	TJ2017	2017	VI 2.1.1.2.2	MW147625/Pigeon/CHN/TJ2017/2017
MW147626	Pigeon	CHN	BJ2018	2018	VI 2.1.1.2.2	MW147626/Pigeon/CHN/BJ2018/2018
MW271783	Pigeon	CHN	JS-23	2011	VI 2.1.1.2.2	MW271783/Pigeon/CHN/JS-23/2011
MW271784	Pigeon	CHN	ZJ-03	2013	VI 2.1.1.2.2	MW271784/Pigeon/CHN/ZJ-03/2013
MW271785	Pigeon	CHN	JS-01	2015	VI 2.1.1.2.2	MW271785/Pigeon/CHN/JS-01/2015
MW271786	Pigeon	CHN	AH-06	2016	VI 2.1.1.2.2	MW271786/Pigeon/CHN/AH-06/2016
MW271787	Pigeon	CHN	JS-09	2016	VI 2.1.1.2.2	MW271787/Pigeon/CHN/JS-09/2016
MW271788	Pigeon	CHN	GS-12	2017	VI 2.1.1.2.2	MW271788/Pigeon/CHN/GS-12/2017
MW271789	Pigeon	CHN	AH-01	2020	VI 2.1.1.2.2	MW271789/Pigeon/CHN/AH-01/2020
MW271790	Pigeon	CHN	JS-01	2020	VI 2.1.1.2.2	MW271790/Pigeon/CHN/JS-01/2020
MW271791	Pigeon	CHN	JS-06	2020	VI 2.1.1.2.2	MW271791/Pigeon/CHN/JS-06/2020
MW271792	Pigeon	CHN	JS-07	2020	VI 2.1.1.2.2	MW271792/Pigeon/CHN/JS-07/2020
MZ101342	Pigeon	UKR	Petruk	2015	VI 2.1.1.2.2	MZ101342/Pigeon/UKR/Petruk/2015
MZ223436	Pigeon	CHN	JS-02	2020	VI 2.1.1.2.2	MZ223436/Pigeon/CHN/JS-02/2020
MZ223437	Pigeon	CHN	HN-01	2020	VI 2.1.1.2.2	MZ223437/Pigeon/CHN/HN-01/2020
MZ223438	Pigeon	CHN	HN-02	2020	VI 2.1.1.2.2	MZ223438/Pigeon/CHN/HN-02/2020
MZ277620	Pigeon	CHN	0044	2019	VI 2.1.1.2.2	MZ277620/Pigeon/CHN/0044/2019
MZ277621	Pigeon	CHN	0054	2019	VI 2.1.1.2.2	MZ277621/Pigeon/CHN/0054/2019
MZ277622	Pigeon	CHN	0072	2019	VI 2.1.1.2.2	MZ277622/Pigeon/CHN/0072/2019
MZ306222	Turtle_dove	CHN	GX11	2018	VI 2.1.1.2.2	MZ306222/Turtle_dove/CHN/GX11/2018
MZ363634	Pigeon	CHN	GX03	2017	VI 2.1.1.2.2	MZ363634/Pigeon/CHN/GX03/2017
MZ395248	Pigeon	CHN	GX04	2017	VI 2.1.1.2.2	MZ395248/Pigeon/CHN/GX04/2017
MZ395249	Turtle_dove	CHN	GX05	2017	VI 2.1.1.2.2	MZ395249/Turtle_dove/CHN/GX05/2017
MZ395250	Turtle_dove	CHN	GX06	2017	VI 2.1.1.2.2	MZ395250/Turtle_dove/CHN/GX06/2017
MZ395251	Pigeon	CHN	GX07	2017	VI 2.1.1.2.2	MZ395251/Pigeon/CHN/GX07/2017
MZ400779	Turtle_dove	CHN	GX08	2017	VI 2.1.1.2.2	MZ400779/Turtle_dove/CHN/GX08/2017
MZ400780	Turtle_dove	CHN	GX09	2017	VI 2.1.1.2.2	MZ400780/Turtle_dove/CHN/GX09/2017
MZ405131	Spotted_dove	CHN	GX07	2018	VI 2.1.1.2.2	MZ405131/Spotted_dove/CHN/GX07/2018
MZ405137	Pigeon	CHN	GXD28	2020	VI 2.1.1.2.2	MZ405137/Pigeon/CHN/GXD28/2020
MZ620686	Spotted_dove	CHN	GX08	2018	VI 2.1.1.2.2	MZ620686/Spotted_dove/CHN/GX08/2018
MZ620687	Spotted_dove	CHN	GX09	2018	VI 2.1.1.2.2	MZ620687/Spotted_dove/CHN/GX09/2018
MZ620688	Turtle_dove	CHN	GX10	2018	VI 2.1.1.2.2	MZ620688/Turtle_dove/CHN/GX10/2018

Accession no.	Host	Country	Strain	Year	Lineage	Label
MZ620690	Spotted_dove	CHN	GX15	2018	VI 2.1.1.2.2	MZ620690/Spotted_dove/CHN/GX15/2018
MZ620691	Pheasant	CHN	GX13	2018	VI 2.1.1.2.2	MZ620691/Pheasant/CHN/GX13/2018
MZ620692	Quail	CHN	GX14	2018	VI 2.1.1.2.2	MZ620692/Quail/CHN/GX14/2018
MZ620693	Turtle_dove	CHN	GX16	2018	VI 2.1.1.2.2	MZ620693/Turtle_dove/CHN/GX16/2018
MZ620694	Turtle_dove	CHN	GX17	2018	VI 2.1.1.2.2	MZ620694/Turtle_dove/CHN/GX17/2018
MZ620695	Pheasant	CHN	GX04	2019	VI 2.1.1.2.2	MZ620695/Pheasant/CHN/GX04/2019
MZ620696	Spotted_dove	CHN	GX06	2019	VI 2.1.1.2.2	MZ620696/Spotted_dove/CHN/GX06/2019
MZ620698	Turtle_dove	CHN	GX11	2019	VI 2.1.1.2.2	MZ620698/Turtle_dove/CHN/GX11/2019
ON637881	Turtle_dove	TWN	C239	2021	VI 2.1.1.2.2	ON637881/Turtle_dove/TWN/C239/2021
ON645962	Pigeon	MKD	230	2008	VI 2.1.1.2.2	ON645962/Pigeon/MKD/230/2008
ON645964	Pigeon	MKD	232	2008	VI 2.1.1.2.2	ON645964/Pigeon/MKD/232/2008
ON645965	Pigeon	MKD	234	2007	VI 2.1.1.2.2	ON645965/Pigeon/MKD/234/2007
ON645969	Pigeon	MKD	1503	2007	VI 2.1.1.2.2	ON645969/Pigeon/MKD/1503/2007
ON645970	Pigeon	MKD	962	2008	VI 2.1.1.2.2	ON645970/Pigeon/MKD/962/2008
ON645971	Pigeon	MKD	1501	2007	VI 2.1.1.2.2	ON645971/Pigeon/MKD/1501/2007
ON645972	Pigeon	MKD	2810	2010	VI 2.1.1.2.2	ON645972/Pigeon/MKD/2810/2010
ON939087	Pigeon	CHN	210818	2018	VI 2.1.1.2.2	ON939087/Pigeon/CHN/210818/2018
ON986198	Chicken	CHE	V043-1	2022	VI 2.1.1.2.2	ON986198/Chicken/CHE/V043-1/2022
ON986199	Chicken	CHE	V043-3	2022	VI 2.1.1.2.2	ON986199/Chicken/CHE/V043-3/2022
ON986200	Chicken	CHE	V043-7	2022	VI 2.1.1.2.2	ON986200/Chicken/CHE/V043-7/2022
ON986201	Chicken	CHE	V043-9	2022	VI 2.1.1.2.2	ON986201/Chicken/CHE/V043-9/2022
ON986206	Pigeon	CHE	V051-1	2022	VI 2.1.1.2.2	ON986206/Pigeon/CHE/V051-1/2022
KJ544861	Human	NLD	NL-03	2003	VI 2.1.1.2.2	KJ544861/Human/NLD/NL-03/2003
JX901120	Pigeon	BEL	05-03936-8	2005	VI 2.1.1.2.2	JX901120/Pigeon/BEL/05-03936-8/2005
HM063425*	Pigeon	CHN	P4	XXXX	VI 2.1.1.2.1	HM063425/Pigeon/CHN/P4/XXXX

*Strain used as outgroup for rooting tree.
